# Analysis of quantitative metrics for assessing resilience of human-centered CPPS workstations

**DOI:** 10.1038/s41598-023-29735-1

**Published:** 2023-02-20

**Authors:** Tanel Aruväli, Matteo De Marchi, Erwin Rauch

**Affiliations:** Free University of Bozen-Bolzano, Piazetta del Università 1, 39100 Bozen-Bolzano, Italy

**Keywords:** Mechanical engineering, Electrical and electronic engineering

## Abstract

Manufacturing companies’ preparedness level against external and internal disruptions is complex to assess due to a lack of widely recognized or standardized models. Resilience as the measure to characterize preparedness against disruptions is a concept with various numerical approaches, but still lacking in the industry standard. Therefore, the main contribution of the research is the comparison of existing resilience metrics and the selection of the practically usable quantitative metric that allows manufacturers to start assessing the resilience in digitally supported human-centered workstations more easily. An additional contribution is the detection and highlighting of disruptions that potentially influence manufacturing workstations the most. Using five weighted comparison criteria, the resilience metrics were pairwise compared based on multi-criteria decision-making Analytic Hierarchy Process analysis on a linear scale. The general probabilistic resilience assessment method Penalty of Change that received the highest score considers the probability of disruptions and related cost of potential changes as inputs for resilience calculation. Additionally, manufacturing-related disruptions were extracted from the literature and categorized for a better overview. The Frequency Effect Sizes of the extracted disruptions were calculated to point out the most influencing disruptions. Overall, resilience quantification in manufacturing requires further research to improve its accuracy while maintaining practical usability.

## Introduction

Disruptions and adverse events in the world may influence and interrupt the production processes in a manufacturing company for months and even years. The events of severe disruptions are often related to external changes which are out of manufacturers’ sphere of influence. Manufacturers are impressionable from external changes, consequently without an ability to counteract the source of change. Recent severe disruptions such as the spread of Covid-19, Suez Canal blockage by cargo ship, the war in Ukraine and geopolitical sanctions had and still have severe influences internationally. External disruptions are more complicated to predict as they often occur unexpectedly compared with manufacturing internal adverse events. The behavior of internal disruptions is more predictable and controllable, but without a systematic approach to managing them, the consequences can be even severer. Although many causes of internal disruptive events are known (worker absence, machinery breakdown, misinformation, lack of information, outage of material or instruments, etc.) the overall preparedness for their actual occurrence is complicated to estimate. Complexity is even increased by the fact that the list of disruptive events is not final, and it expands in time due to technological development and overall external environment evolution. Therefore, an unproperly evaluated preparedness level against potential disruptions may have existential consequences for manufacturing companies.

To assess the preparedness level against potential disruptions, the measure of resilience is an important indicator. Resilience is a concept historically more used in social sciences and it has been rather a qualitative measure. In engineering, the feature of resilience has been taken more widely into consideration recently^1^. In manufacturing, during the era of Industry 4.0, the widely used key performance indicator to evaluate everyday production has been productivity which is relatively simple to measure quantitatively. Cyber-Physical Production Systems (CPPS) retrieve various data from manufacturing shopfloors which are mostly used for efficiency-related metrics. The ongoing transition to the era of Industry 5.0 contrarywise, highlights a wider and longer-term view of the manufacturing shopfloor health and the benefits of sustainable manufacturing. Therefore, CPPS have the advantage compared with traditional shopfloors for using retrieved data simultaneously for filling the cap in resilience assessment. Resilience is to encompass all three pillars of Industry 5.0: economic, ecologic, and social aspects. Even so, no widely recognized approach nor equation to measure resilience exists. Therefore, for a specific application, a variety of studies must be reviewed and analyzed to select an optimal tool for the numerical assessment of resilience.

The driver of this research is on one hand lack of a practically usable standard model to measure resilience in manufacturing, on the other hand, a variety of models differing in their inputs, algorithms, and even units of measure. To create more clarity, there is a need for a deeper understanding what are the causes that trigger disruptions and how to quantitatively measure their potential impact on manufacturing resilience. The main research question is formulated as follows:

RQ: How to quantitatively measure resilience in CPPS workstations by considering the most influencing disruptions?

The following three research sub-questions are investigated in this study:

RQ_1_: What are the disruptions potentially influencing manufacturing workstations the most?

RQ_2_: Which quantitative metrics exist to measure the resilience of manufacturing workstations?

RQ_3_: What is the most practically valuable quantitative resilience metric to assess the level of resilience in CPPS workstations?

This work aims to provide an overview of different resilience metrics and to extract the metrics which can be used in the assessment of resilience in manufacturing. More specifically, the quantitative resilience metrics are pointed out which can be efficiently used in the assessment of digital twin supported worker assistance system in manufacturing and in the process of verifying those workstations based on resilience. Additionally, manufacturing-related disruptions are categorized and analyzed. The research is focused on resilience quantification of existent manufacturing systems and excludes system design-related optimization where resilience is evaluated during the design of systems. Further, supply chain related resilience is excluded.

The article is organized as follows: after the overview of the concept of resilience in “Resilience background”; the research questions are formulated and the used methods for literature review and Analytic Hierarchy Process (AHP) analysis process are described in “Methodology”; thereafter in “Results”, the results of the research are presented descriptively and in table format for easier trackability of processes and comparability; the paper is concluded in “Discussion” where the results, future perspectives, and challenges are discussed in more detail.

## Resilience background

### Concept of resilience

Disruptions are the events that cause breakage of resilience. The concept of disruption comprises disturbances and failures^2^, in some contexts disruptions are called shocks^3^ or adverse events^4^. If a deviation from a plan is sufficiently large that the plan must be changed substantially it is called a disrupted situation or disruption^5^. For consistency, the word “disruption” is used in this article where no further specification is needed.

Resilience can be considered belonging to a category of *ilities*^6^*.* The *ilities* are engineering system properties that concern wider system impacts and are not considered primary functional requirements in contrast to reliability, robustness, and durability. Resilience supports other *ilities* such as safety, sustainability, quality, and flexibility^7^.

The researchers have provided different definitions of the term “resilience”. In general, common positions in definitions are that resilience includes three focal components: (i) an ability to absorb the impact of disruptions (absorption), (ii) adaptation to disruptions (adaptation), and (iii) recovery to its normal regime (restoration). The pathfinder of defining the term of resilience as a property of a system is Holling who expressed, resilience determines the persistence of relationships within a system and is a measure of the ability of these systems to absorb changes of state variables, driving variables, and parameters, and still persist^8^. Gu et al.^9^ defined resilience as the ability of a system to withstand potentially high-impact disruptions, and it is characterized by the capability of the system to mitigate or absorb the impact of disruptions, and quickly recover to normal conditions. Whereas Gasser et al.^10^ pointed out modern understanding of resilience as a process under which the observed system undergoes in response to a disruption quantified in terms of a measure of system performance and its evolution over the system response time after an event. Romero et al.^11^ combined the definitions of resilience and smartness and defined the concept Smart Resilient Manufacturing System as an agile and flexible/reconfigurable system that uses smart sensor systems and descriptive, predictive, and prescriptive analytics techniques to collect and analyze in real-time operational and environmental data to anticipate, react, and recover from a disruption. While Yoon et al.^12^ proceeded with resilience definition in engineering as the ability of a component or a system to maintain its required functionality by resisting and recovering from adverse events.

Hence, resilience is a multifactorial concept, it can be resolved by several factors which in a more focused way characterize the features of it. According to Hu, the resilience of an engineering system consists of three key elements: reliability, vulnerability, and recoverability^13^, while Lim et al.^14^ have replaced the element of vulnerability with redundancy. The system’s capability of maintaining its functions and structure in the situation of internal or external changes is part of resilience^15^. If passively reliable equals vulnerable then adoptively reliable equals resistant^16^. Requirements such as functionality, rapidity, and resourcefulness are also brought out as properties of resilience^17^. Additionally, resilience engineering factors to consider in manufacturing are flexibility, redundancy, and fault-tolerant^18^.

There are other engineering system properties as reliability and stability principally differentiate and must be distinguished from resilience. Reliability measures the continued success of a system, while resilience measures the insensitivity of the system to disruptions^19^. Whereat fault isolation is an important aspect in achieving internal reliability^20^, which in turn increases resilience. Holling pointed out a principal difference between resilience and stability^8^, stability is an ability of a system to return to equilibrium with the least fluctuation after a temporary disruption. A system can have high resilience, but still fluctuate greatly and have low stability.

Preventive maintenance and redundancy are two main methods for disruptions management^21^. In manufacturing, redundancy is often related to having a backup machineries and workforce. However, redundancy can be also achieved at the system management level as informational redundancy, for instance in knowledgeable implementation of Enterprise Resource Planning^22^. A high level of redundancy also increases a system’s life cycle cost^16^ and cannot be a sustainable solution. Another widely practiced method for disruptions management is quantitative risk assessment which mainly focuses on the pre-failure scenarios^23^ but can offer a base for the assessment of resilience if supported with advanced analytics^4^. It is accepted that every risk cannot be foreseen but it must be rather learned to adapt and manage risks in a way to minimize the impact on systems^24^.

### Quantification of resilience

**Figure 1:**
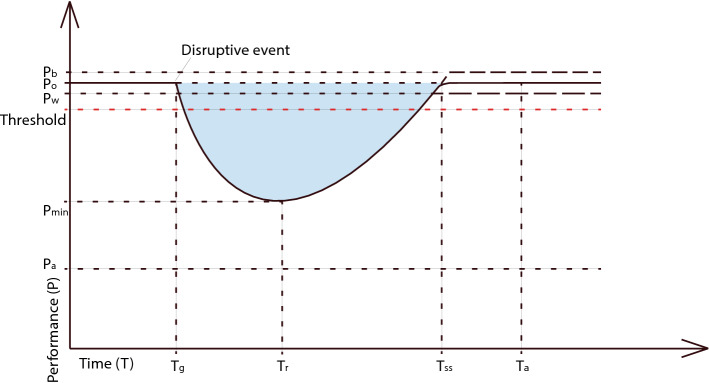
Performance dependence from disruptive event and recovery action (adapted from Refs.^25^ and ^26^).

Resilience is a time-dependent phenomenon. In a system, after an event of a disruption (T_g_), the performance of the system starts to decrease. The performance starts to increase again after the event of recovery action (T_r_) and the performance increases until the system achieves its steady state (T_ss_). Whereas the recovered performance can recover to its original level (P_0_), to have a shortfall (P_w_) or to have a growth (P_b_). P_min_ represents the minimum level of performance over disruption. In the time dimension, relative to the occurrence of a disruptive event, three phases are distinguished: pre-disaster phase (− T_g_), during disaster phase (T_g_–T_r_) and post-disaster phase (T_r_–∞)^17^. Maximum acceptable recovery time (T_a_) and minimum acceptable performance level (P_a_) are proposed, below which operations are presumed to shut down^25^. The blue area (Fig. 1) represents total loss of performance due to disruption.

In manufacturing systems, resilience is mostly measured as a technical attribute as loss of productivity, it is also named performance loss or loss of throughput. Resilience is an abstract concept and expressing its value by loss of manufacturing throughput only is a rather simplified approach. Counterweight, Bruneau et al.^27^ proposed four dimensions of community resilience—(1) technical, (2) organizational, (3) social, and (4) economic that are so far not approached in a socio-technical system as human-centered workstation.

To understand the purposes and functions behind the measuring, the measuring framework has been developed. Linkov et al.^4^ have created a resilience matrix to provide guidelines based on what resilience metrics can be developed to measure overall system resilience. In this matrix system domains (physical, information, cognitive, social) across an event management cycle of resilience functions (plan/prepare, absorb, recover, adapt) are mapped and described.

**Figure 2:**
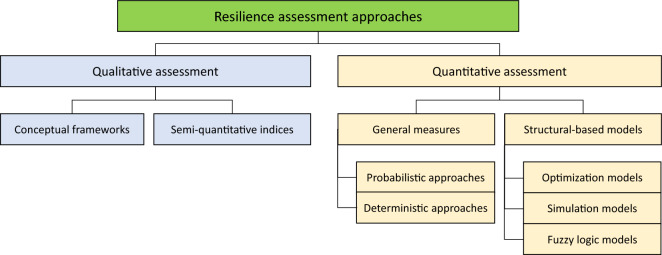
Classification of resilience assessment methodologies (redrawn from Ref.^30^).

In a large scale, resilience assessment approaches can be divided into qualitative and quantitative (Fig. 2). The quantitative measures are typically not optimal, possible, or desirable. Therefore, semi-quantitative measures are often used in the assessment. Quantitative^28^ assessment is expressed in numerical values and is stated in measurement-related specific units. In resilience assessment, these can be divided into general measures and structural-based models. Semi-quantitative^29^ assessment uses qualitative categories that assign numeric values which are thereafter calculated as indices, the assessment often needs an expert opinion. The least precise method is qualitative conceptual framework assessment which contributes notion of resilience.

The methods as observation, interview, expert opinion, and focus group can be considered as main qualitative data collection methods. The data is often collected in different formats as audio, video, and text. In supply chain resilience assessment Nikookar et al.^31^ used questionnaire in their case study for suppliers evaluation. Garcia et al.^32^ analyzed resilience qualitatively in ICT based network systems where they compared fidelity on different emulation testbeds and recommended to observe system behavior and trend inside the system. For qualitative data representation, quantitative analytics can be applied as a semi-quantitative approach. Such a scenario was used by Eljaoued et al.^33^ where qualitative functional resonance analysis method with numerical approach was used in socio-technical system resilience assessment.

Without any numerical basis for assessing resilience, it is complicated to monitor and track the improvements. Numerical measuring allows targets to be established and set clear goals for improvement. After Youn et al.^16^ pioneered a way to measure the engineering design resilience quantitatively, more explicit models have been developed which additionally consider various characteristics as explicit temporal aspects^26^ and sensor faults^34^.

Research of resilience metrics in manufacturing rather focuses on resilience design methods^35,36^, than assessment and validation of existing systems. Even though, resilience metrics assessment methodology based on experimental design and statistical analysis has been proposed to validate the metrics^37^. Still, some resilience assessment case studies have been recently conducted in the industry overall. Hybrid simulation software Anylogic was used to evaluate the impact of disruptions in the cork industry, different disruption scenarios were analyzed and numerically represented^38^. According to a recent (2021) review on resilience in Cyber-Physical System^39^, from 390 relevant articles 32 papers were in the domain of manufacturing and 24 of the papers had resilience metrics related approach. Still, none of the 32 papers in the domain of manufacturing had resilience metrics approach. Thus, resilience metrics in CPPS have not been in the focus of recent research.

### Discussion of existing literature

Resilience is abstract concept with many definitions. Which properties it exactly gathers and what is their allocation are still under scientific discussion. The related characteristics mainly brought out in various approaches are reliability, redundancy, flexibility, sustainability, and maintaining of functionality. Resilience is rather imagined as dynamic temporal measure that needs constant input for its quantification. What is agreed is that resilience is a valuable indicator, and its quantification enables companies to assess their long-term success and to be better prepared for disruptions. Despite many approaches to quantify resilience in manufacturing, no industry standard metric is recognized so far.

Applying of resilience metrics in CPPS have not been in the research focus. Although CPPS have higher potential to contribute with numerical inputs to the resilience assessment, these have not been in standalone observation in resilience perspective so far. However, as explained earlier, many overall engineering and manufacturing resilience metrics have been proposed. The further analyze of these metrics’ gains in recognition of CPPS suitable metrics. Their deeper analyze may come up with most practically suitable solution to be considered to become a recognized standard.

## Methodology

**Figure 3:**
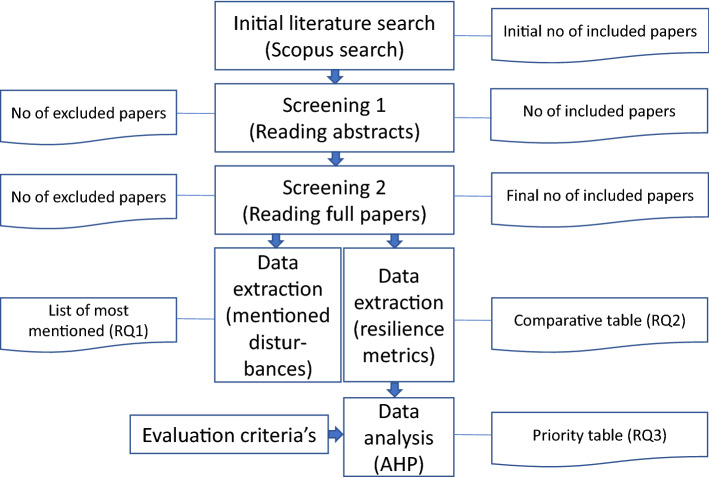
Main methodology of the research.

This study adopts a descriptive literature review methodology (a) to find the most common disruptions in manufacturing and (b) to find and compare the resilience metrics which can be used in the resilience quantification of manufacturing workstations. For the investigation of mentioned disruptions, a quantitative meta-summary was used. For resilience metrics comparison, multi-criteria decision-making AHP analysis in linear scale was applied. The main sequence of research methodologies is presented in Fig. 3.

### Literature search and evaluation for inclusion

The literature review was conducted to investigate the research questions. Scopus database was selected as a source for literature as it is internationally recognized, in addition, it contains a large number of publications from the area of engineering sciences. The search was performed on the 9th of March 2022. Search query string (TITLE-ABS-KEY ("resilience assessment" OR "resilience metric*" OR "resilience measure*" OR "resilience analysis" OR "resilience quantification" OR "evaluating resilience") AND ALL ("manufacturing plant" OR "manufacturing system" OR "resilient production" OR "engineering resilience") AND NOT ALL (ecosystem) AND NOT ALL (seismic)) OR (TITLE-ABS-KEY (resilience AND (assessment OR metrics)) AND TITLE-ABS-KEY (manufacturing)) AND (LIMIT-TO (LANGUAGE, "English")) AND (EXCLUDE (LANGUAGE, "Spanish") OR EXCLUDE (LANGUAGE, "Italian")) was chosen for literature search. The query string was divided into three parts (divided by brackets). The first part focuses on publications regarding the resilience quantitative approach, while extra to manufacturing field engineering resilience (as manufacturing systems and workstations are part of it) was also included. Whereas manufacturing and engineering-related keywords were searched from all text to receive a wider scope of results. In turn, it was needed to exclude some query terms from environmental topics (ecosystem, seismic) to stay in scope. The second part of the query string covers resilience in manufacturing overall, these were only searched from abstracts, keywords, and titles as these keywords are more comprehensive. Therefore, the terms “assessment” and “metrics” were allowed to be separated from the term “manufacturing”. The third part limits results to the English language only. Nevertheless, Italian and Spanish needed to be separately excluded to receive only results in English. To cover possible disruptions over time, no time limit was set. Secondary documents and patents were excluded.

The first screening included the reading of abstracts. If the abstract supplied no sufficient information, the content of a publication was also overlooked. Only publications with a focus on topics such as manufacturing, industry resilience generally and engineering system resilience were approved for inclusion criteria. Manufacturing-related publications were included, except food and oil industry. Additionally, manufacturing-related product design, risk analysis, and supply chain publications were excluded. Whereas system engineering publications were included only if these were focused on resilience metrics. In addition, full proceedings and duplicates were excluded. Moreover, inaccessible publications were excluded as well.

The second round of screening consisted of reading the papers. Included were only papers mentioning disruptions (including disruptive events, adverse events, shocks, and detrimental events) in manufacturing or contributing resilience metrics for manufacturing. Resilience design related documents were excluded.

### Disruptions

For the investigation of mentioned disruptions, a review methodology quantitative meta-summary^40^ was applied. First, descriptive expressions which could be viewed as disruption causes or disruption modes were extracted. Thereafter, expressions describing the same disruption with different words were identified and rephrased to describe their common meaning. Frequency Effect Sizes of mentioned disruptions and Intensity Effect Sizes^41^ of articles were calculated and analyzed.

As papers focusing on supply chain were excluded, also general supply chain disruptions pointed out in included articles as a cause of disruptions were not separately categorized to avoid distortions in results. Supply chain disruptions caused specific consequences more related to manufacturing processes were listed instead.

The list of mentioned disruptions was divided into external and internal disruptions. External disruptions were divided into subcategories based on STEEPLE (Social, Technical, Economic, Environmental, Political, Legal, and Ethical), which has been developed for the analysis of key system elements in manufacturing^42^. Still, some STEEPLE categories were united having a close relationship with each other in the resilience context (social and ethical; political and legal; technical and economic), while technical was replaced with technological.

For the classification of internal disruptions, the model of the Automation Pyramid^43^ was taken as a basis. Although, modern workstations are highly automated, still human in the loop is often present. Therefore, the human aspect was included for levels 2–4, whereas operator-related disruptions were covered in the second level. Thus, internal disruptions were divided into five subcategories as follows: field, control, operator, planning, and management.

Some of the mentioned disruptions can reflect both, external and internal disruptions. In this case, the following characteristics were analyzed. At first, the category with stronger influence was identified (e.g., internal social unrest is with higher influence). Secondly, it was asked if a company possesses direct and fast influence over the disruption. If the answer was negative, still external category was selected (e.g., availability of investment capital). If mentioned disruption has consequences in at least two subcategories, the source subcategory was detected (e.g., Covid-19 source category is social, although political and economic aspects were also present). In categorizing internal disruptions, the automation pyramid lower level was preferred (e.g., the material shortage was categorized as management-related disruption, while material delay and poor material supply influence post-planning activities and was categorized accordingly).

Different expressions for the same or similar disruption were counted as the same type of disruption (“deterioration in quality output” and “quality flaws” were both counted as “output quality flaws”). If a more specific reason was also brought out, the disruption was counted separately and not listed as a related general disruption (“bias of the pallet (tire treads exceed on the side)” refers to quality but was listed separately).

### Resilience metrics comparison

Multi-criteria decision-making tool, standard linear scale AHP^44^ was used to compare extracted resilience metrics’ practical value in digital twin supported worker assistance system in manufacturing. For AHP process parameters set up and calculation, an online tool AHP-OS^45^ was used.

Comparison criteria were chosen as the most important characteristics for real-life practical usability:

- Feasibility—not only theoretical, supported with case studies or examples, real-life tested and repeatable.
- Relevance—suitability for this specific application: digital twin supported human-centered assembly station.
- Accuracy—more measuring or experiment based and less probabilistic or expert opinion driven.
- Comprehensiveness—takes into account a wide variety of possible disruptions, including different agents such as humans and technology-related components as well as the external and internal types of disruptions.
- Comparability—comparability levels: comparable with the same type of workstations, comparable with a different type of workstations in the same plant, comparable between different companies.

One level hierarchy of AHP analysis was used. Comparison criteria weights (Table 1) were derived by pairwise comparison of criteria on a ratio scale from 1 to 9 (scale defined by the software). The scale of comparison characterizes the intensity of importance as follows: 1—equal importance (two elements contribute equally to the objective), 5—moderate importance (experience and judgment moderately favor one element over another, 9—strong importance (experience and judgment strongly favor one element over another). The values between these numbers characterize intermediary levels accordingly.

**Table 1:** Weights of comparison criteria for AHP.

Criterion	Priority (%)
Feasibility	38.1
Relevance	38.1
Accuracy	3.2
Comprehensiveness	8.0
Comparability	12.6

**Figure 4:**
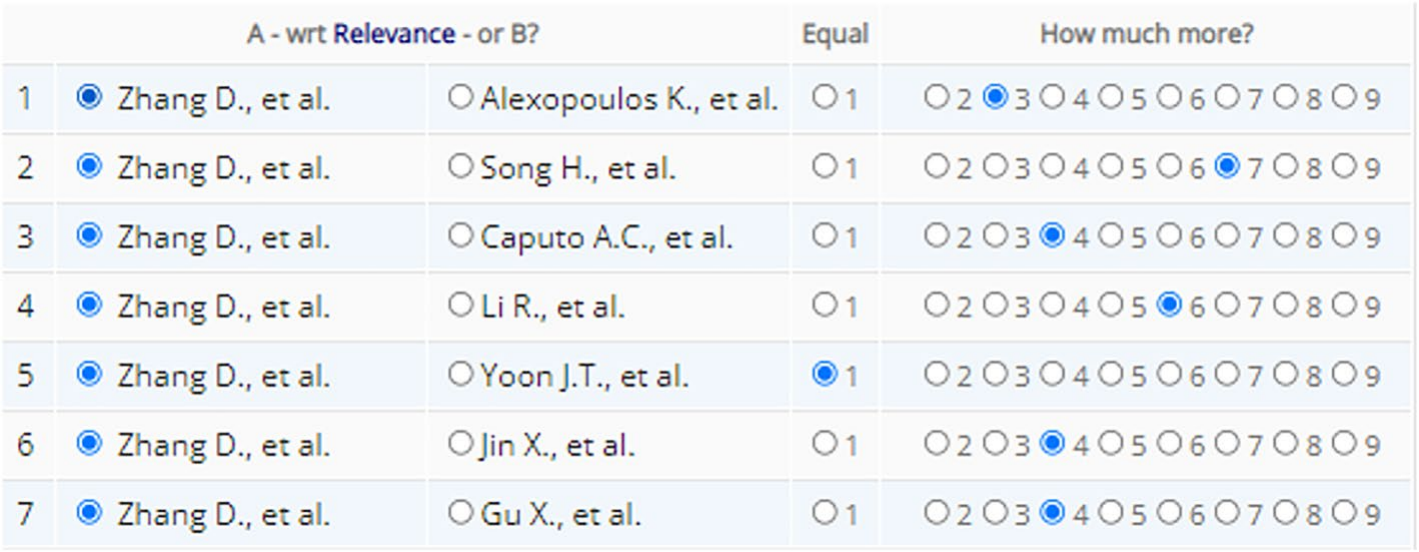
Pairwise comparison of Zhang et al. proposed resilience metric against other selected metrics under criteria of relevance (screenshot from AHP-OS software).

**Figure 5:**
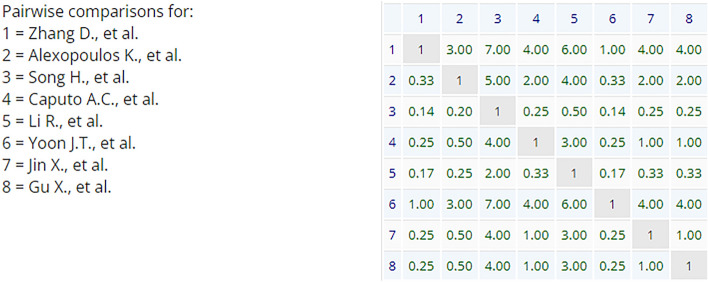
Decision matrix for the criterion of relevance (screenshot from AHP-OS software). The row no. 1 and the column no. 1 correspond with Fig. 4 comparison results.

For resilience metrics pairwise comparison the previously described AHP analysis in scale from 1 to 9 was used again in the same way. The process of comparison started with the decision which metric is more valuable under specific criterion and secondly how much more in given scale (Fig. 4). The comparison was made by two authors together on a consensus basis. In cases the consensus could not be found between these authors, an additional expert (in the field of sustainability and resilience) opinion was asked. In each pairwise comparison, consistency ratio was calculated for inconsistency assessment. As the comparison result was a collective decision, some inconsistency is admissible. After the comparison of all pairs, the decision matrix was received (Fig. 5).

**Figure 6:**
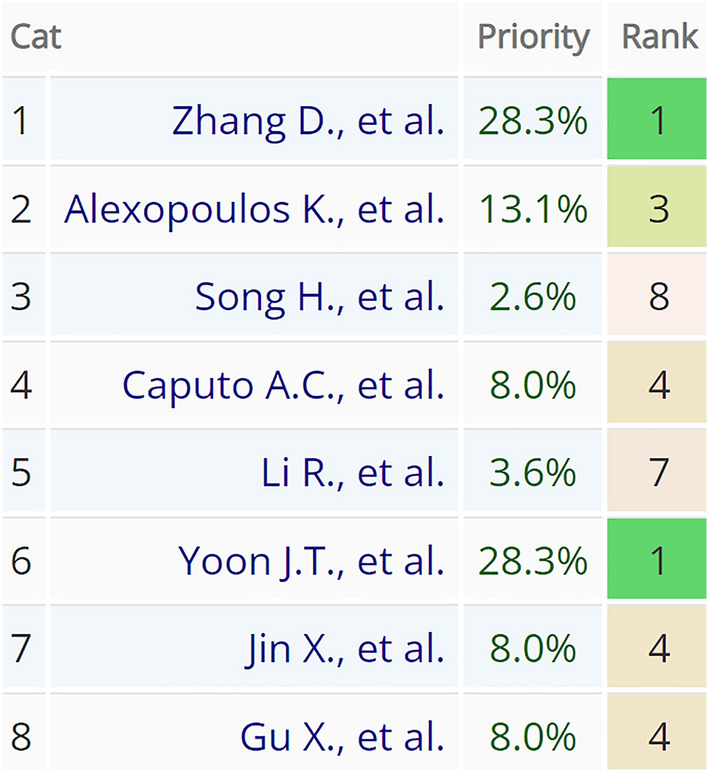
Consolidated priority scores under the criterion of relevance comparison (screenshot from AHP-OS software).

Based on decision matrixes, resilience metrics consolidated priorities were calculated under each comparison criterion (Fig. 6). Subsequently, each resilience metric consolidated priority scores were summed to receive the final AHP analysis results. To illustrate the process, only criterion of relevance related figures are presented in the article. The rest of the pairwise comparisons can be found in Supplementary Fig. S1.

## Results

### Literature search and screening

**Figure 7:**
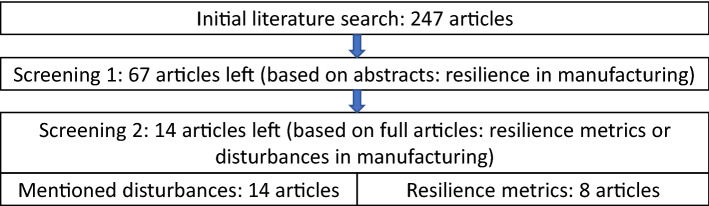
Literature review in numbers.

Based on the literature review, quantification of resilience in manufacturing (excluding design of manufacturing systems) processes was provided in 8 articles and specific disruption causes were mentioned in 14 articles. Screening of review results is presented in Fig. 7. More detailed screening details can be found in Supplementary Table S1.

90% of initial literature search articles were published in 2013 or later, whereas over 50% of articles were published within the last 3 years. Hence state-of-the-art articles were found without limiting the year of publications. The first screening excluded 180 articles, mostly from other engineering areas. Namely (listed decreasingly) supply chain, infrastructure, power engineering, environment, medical engineering, economy, construction engineering, material engineering, product design, and others (Supplementary Table S1). The second screening excluded 53 articles proposing no relevant resilience metrics nor mentioning specific manufacturing-related disruptions. The second screening revealed that relevant manufacturing-based resilience metrics were proposed in 12% of manufacturing resilience related articles, while disruptions (disruption causes and disruption modes) were mentioned in 21% of the articles. Whereas all articles in which resilience metrics were proposed, the disruptions were also mentioned.

### Manufacturing influencing disruptions

The findings of disruption mentions were found in articles published from the year 2015 to 2021. In newer articles the average number of findings is smaller, resulting in 57% of findings from the year 2016. A total of 86 disruption mentions were extracted (Table 2). The list of disruptions is highly influenced by two articles, a total of which provide 50% of the findings. The article^46^ with the highest intensity effect size of all findings (22 findings) is general smart manufacturing systems based and refers to agile manufacturing when listing disruptions. The second highest intensity effect size of all findings (21 findings) article^47^ is also general by focusing on re-distributed manufacturing.

**Table 2:** List of articles with findings of disruption mentions and their intensity effect sizes of all findings (86 findings) and intensity effect sizes of findings with frequency effect sizes > 20% (7 findings—Table 4).

Authors of the article	Year	No. of findings	Intensity effect size of all findings	Intensity effect size > 20%
Peng et al.^48^	2021	1	0.01	0.14
Zhang et al.^49^	2021	12	0.14	0.57
Alexopoulos et al.^50^	2021	3	0.03	0.43
Latsou et al.^36^	2021	3	0.03	0.14
Song et al.^19^	2020	1	0.01	0
Okorie et al.^51^	2020	3	0.03	0.43
Diaz-Elsayed et al.^52^	2020	2	0.02	0.14
Caputo et al.^53^	2019	1	0.01	0.14
Li et al.^54^	2019	5	0.06	0.29
Yoon et al.^12^	2017	1	0.01	0
Kibira et al.^46^	2016	22	0.26	0.29
Jin et al.^55^	2016	6	0.07	0.57
Freeman et al.^47^	2016	21	0.24	0.29
Gu et al.^9^	2015	5	0.06	0.43
Total		86	1	

Of 86 mentions, 58 disruptions were interpreted as unique: 27 as external and 31 as internal (Table 3). Mentioned disruptions were sometimes general as machine fault, but often specific as solenoid valve disfunction or dye stripping fault. Whereby, in some articles, general disruptions were named and more specific reasons were brought out in addition. Regardless of their comprehensiveness level, all mentioned disruptions were analyzed equally to receive a natural view of mentioned disruptions.

**Table 3:** Mentioned unique disruptions based on category.

Disruption category	No. of unique disruptions	Disruptions
External	27	
Environmental	8	Fire, earthquake, flood, natural disaster, hurricane, extreme climatic events, shifts in weather patterns, climate change
Political and legal	3	Changing regulations, sudden changes in political landscape, role of global non-profit and philanthropy organizations
Social and ethical	5	Covid-19, pandemics, terrorism, changing demographics, shocks that change ethical stances
Economic and technological	11	Modifications in the demanded volume of product(s), power or water outages, changes in availability of materials or parts, changes in cost of materials or parts, availability of investment capital, economic downturns/upturns, globalization, future markets, dynamic of technology and innovation, supplier bankrupt, uncertainty and dynamicity environment
Internal	31	
Field	6	Machine faults / machine breakdown, sensor faults, screwdriving device wear, vacuum absorption, solenoid valve malfunction, dye stripping fault
Control	2	Control network fault, system connection failure
Operator	7	Fluctuation of processing time, labor tiredness, output quality flaws, bias of the pallet, labor shortage, social unrest, changes in workforce
Planning	9	Scheduled maintenance, delayed material supply, changed product routing, customer order reprioritization, rush order, order change, mass customization, poor material supply, order cancellation
Management	7	Product returns, new equipment installation, new production line configuration, new software installation, new product introduction, changes in business ownership, changed product line
Total	58	

A total of 7 disruptions (hereinafter effective disruptions) received mentions from > 20% of articles. The most frequently mentioned disruptions were “natural disaster, “covid-19” and “changes in availability of materials and parts” (Table 4). From these disruptions, in some case-specific disruptions and their higher-level general disruptions were represented (covid-19 belonging under pandemics and earthquake belonging under natural disaster).

**Table 4:** Effective findings (frequency effect size over 20%) of disruptions.

Subcategory	Disruption	No. of mentions	Frequency effect size
External			
Environmental	Natural disaster	5	0.36
Environmental	Earthquake	3	0.21
Social and ethical	Covid-19	5	0.36
Social and ethical	Pandemics	3	0.21
Social and ethical	Terrorism	3	0.21
Economic and technological	Changes in availability of materials or parts (shortage)	5	0.36
Internal			
Field	Machine faults/breakdown	3	0.21

Intensity effect size > 20% (Table 2) characterizes the percentage of effective findings (mentioned > 20% of articles) in a certain article from all effective findings. Intensity effect size shows the importance and trustfulness of the article regarding the findings.

Unique external and internal disruptions show approximately equal mentions, 27 and 31 accordingly. However, frequency effect size shows more disruptions in external than internal, 6 vs 1 disruption. This denotes, the internal category includes more single-mentioned disruptions. It reflects many internal disruptions being a company or narrow manufacturing field or system specific whilst external disruptions are more common overall in manufacturing companies. For external disruptions, the category economic and technological received the most mentions and the most unique mentions. For internal disruptions, planning-related disruptions were the most mentioned (both, total mentions and unique mentions). In some subcategory disruption lists, general and more precise disruptions (belonging under the same general disruption) are both represented. This illustrates mostly the disruptions which were more often mentioned (natural disasters, covid-19, machine faults/breakdown). Thus, regardless of modest sampling data, more often mentioned disruptions can be confirmed are trustful as they were supported by both, general and specific disruption reasons.

### Resilience metrics review and analysis

Eight articles with relevant resilience metrics (Table 5) were identified and researched to compare their features in five categories using AHP analysis. As a result, the methodology Penalty of Change (POC) was evaluated as the most suitable and practically usable quantitative resilience assessment metric for digitally supported human-centric workstations in SMEs.

**Table 5:** Comparison of resilience metrics based on quantification methods, strengths, and limitations.

Article	Methods	Strengths	Limitations
Zhang et al.^49^	Based on max-plus algebra	Uses a digital twin based automatic resilience evaluation system	It focuses on system internal disruptions only. Needs historical datasets regarding fault modes to evaluate resilience
Alexopoulos et al.^50^	Generic algorithm (probabilistic)	It combines both technological and economical terms and requires no large and complex amounts of data for calculations. Disruptions are observed as an ignition for system changes. Production-related aspects, such as varying types of products, operational status, and varying demand, can be described and utilized in a common context	Dependent on disruptions occurrence probabilities estimation
Song et al.^19^	Fuzzy logic and generic algorithm	The resilience model can be also used to solve other combination problems. Considers also cost and reputation factors	Focus on cloud manufacturing only
Caputo et al.^53^	Generic algorithm (deterministic)	Step by step description of the process explained. Based on resilience, economic loss is calculated. Manufacturing was observed as the quality of service to estimate resilience	Addresses full plant processes and systems, rather than one workstation. No experiment or case study was included
Li et al.^54^	DMEA and Monte Carlo simulation based (deterministic)	Considers different types of resilience behaviors based on specific disruption	Only considers internal disruptions and needs historical datasets for a bottom-up approach
Yoon et al.^12^	General algorithm (probabilistic)	It is based on the existing resilience equation in which restoration as one component is considered	Focus and description are on sensor false alarms. Systems using prognostics and health management techniques were considered only
Jin et al.^55^	Generic algorithms (probabilistic)	Expands the manufacturing resilience approach by defining 3 resilience metrics: performance loss, performance restoration time, and underperformance time	Case study set-up and resilient calculation not described, but only mentioned
Gu et al.^9^	Generic algorithms (probabilistic)	Expands the manufacturing resilience approach by defining 3 resilience metrics: production loss, throughput settling time, and total underproduction time. Compares resilience to different company policies	Addresses full plant processes and systems, rather than one workstation

#### Descriptive review

Eight resilience metrics are further described below and specifically defined in Table 6 in following related terms: metric symbol and name, formula and symbols definitions, definition of concept resilience, and case study or example use.

**Table 6:** Comparison of resilience metrics based on mathematical formulas, resilience definitions and application examples.

Article	Metric	Formula and symbols	Resilience definition	Example
Zhang et al.^46^	Re	$Re=1/{\int }_{0}^{TS}({TP}^{\text{E}} -TP)dt$ where TS is the total working time of the system, TP^Ē^ is throughput without disruptions and TP is system throughput	Ability to maintain production under disruptions. It measures production losses under disruptions	A digital twin testing platform for smart phone assembly was developed for resilience control of disruptions
Alexopoulos et al.^47^	POC—penalty of possible changes	$POC={\sum }_{i=1}^{D}Pn\left({X}_{i}\right)\mathrm{Pr}\left({X}_{i}\right)$ where D is the number of potential changes, X_i_ is the i-th potential change, Pn(X_i_) is the penalty (cost) of the i-th potential change and, Pr(X_i_) is the probability of the i-th potential change to occur	Changing the system encompasses a potential penalty that may include relevant costs: equipment investment (machines, tooling, etc.), labor training, reprogramming, opportunity costs and others	COVID-19 related pilot case applied to two hypothetical manufacturing systems (3D printing farm vs. injection molding) that produce plastic products for the automotive industry
Song et al.^19^	Q_4_(ψ_i,j_)	$max \,{Q}_{4}({\psi }_{i,j})=\prod_{j=1}^{N}{\psi }_{i,j}(\frac{{\mu }_{i,j}}{M}\sum_{o=1}^{M}{\sigma }_{i,j,m}{en}_{i,j,m}^{Nin,Nout}+\frac{{\nu }_{i,j}}{N}\sum_{p=1}^{L}{\rho }_{i,j,l}{ex}_{i,j,l}^{Nin,Nout})$ where Q_4_(ψ_i,j_) is the resilience of manufacturing service of the i-th candidate of the j-th sub-task; M and L represent the number of endogenous attributes and the number of exogenous attributes, respectively; σ_i,j,m_ and ρ_i,j,l_ represent the weight of each endogenous attributes and the weight of each exogenous attributes, respectively; μ_i,j_ and ν_i,j_ represent the weight of total endogenous attributes and the weight of total exogenous attributes, respectively; en_i,j,m_^(Nin,Nout)^ and ex_i,j,m_^(Nin,Nout)^ are calculated by: ${E}_{A}=\frac{{\int }_{{t}_{a}}^{{t}_{b}}\left|{V}_{A}-{V}_{A}^{E}\right|dt}{max\, {(V}_{A})-min\,({V}_{A})}$ where E_A_ refers to the measured value corresponding to equilibrium A; V_A_ represents real-time measured value of equilibrium A; V_A_^(E)^ represents measured value of equilibrium A in consistent operation; t_a_ and t_b_ represent the establishment time and final time of the inspection. The denominator indicates the value of the maximum change of measured value during the inspection period	Resilience is an attribute of the service, which is used to measure the insensitivity of the system to disturbances	Resilience calculation was one attribute of hybrid resilience-aware global optimization (HRGO) approach. The other HRGO attributes are cost, time, and reliability. Based on HRGO, service composition and optimal selection is tested in a company Chery Automobile Co, where two scenarios are considered and compared
Caputo et al.^50^	Resilience (Caputo et al.)	$Resilience=\frac{1}{{t}_{r}-{t}_{0}}{\int }_{{t}_{0}}^{{t}_{r}}C(t)dt$ where C(t) is nominal capacity, t_0_ is time of disruption and (t_r_–t_0_) is recovery interval	Resilience is a performance measure representing the system ability to survive disruptive events, and the rapidity in restoring system capacity after the disruptive event has occurred	N/A
Li et al.^54^	$\widehat{{R}_{A}}$-Estimation of expected system resilience	${R}_{A}=E\left({R}_{D}\right)\, and\, {\widehat{R}}_{A}=\frac{\sum_{i=1}^{n}{R}_{D,i}}{n}$ where n is the number of the deterministic resilience, R_A_ is the expectation of system resilience and reflects the average resilience of the system, $\widehat{{R}_{A}}$ is an estimate of R_A_ and is calculated by taking the average of the deterministic resilience under different disturbances	Technical resilience refers to the ability of the system to perform at an acceptable level when the disturbance occurs. Economic resilience refers to the capacity of the system to reduce both direct and indirect economic losses resulting from the disturbance	In automatic tire tread handling system subjected to random disturbances, the resilience was evaluated based on 1000 Monte Carlo-based simulation runs and proposed disturbance mode and effects analysis
Yoon et al.^12^	Ψ_FA_—resilience measure that considers false alarms	${\psi }_{FA}=Pr(\hat{H} H)+Pr({E}_{mr}\widehat{F}F)$ where Pr(ĤH) is probabilities of “system normal” operation and Pr(E_mr_ḞF) is system restoration rate	Engineering resilience is the ability of a component or a system to maintain its required functionality by resisting and recovering from adverse events	Two case studies were employed and resilience calculated. The first examines numerical examples and the second studies an electro‐hydrostatic actuator
Jin et al.^55^	RM—resilience metric	Spatial characterization: ${RM}_{i}^{{Sub}_{j}}={RM}_{i}^{{Sub}_{j}}\left({\varphi }_{d};{\varphi }_{S};{\varphi }_{{M}_{1}},{\varphi }_{{M}_{2}},\dots ,{\varphi }_{{M}_{M}};{\varphi }_{{B}_{1}},\dots ,{\varphi }_{{B}_{B}}\right)$ Temporal characterization: ${RM}_{i}^{[T]}=\frac{{\bigcap }_{l=1}^{{N}_{T}}({\varphi }_{{d}_{l}}; {\varphi }_{S}; {\varphi }_{{M}_{1}}, {\varphi }_{{M}_{2}}, ..., {\varphi }_{{M}_{M}}; {\varphi }_{{B}_{1}}, ..., {\varphi }_{{B}_{B}})}{1}$ where ${RM}_{i}^{{Sub}_{j}}$ is the ith resilience metrics for a subsystem j; φ_d_ is the set of parameters that describe the disruption (e.g., starting time, duration, location); φ_S_ is the information related to the system configuration (e.g., serial, parallel, hybrid); ${\varphi }_{{M}_{i}}$ is a set of parameters that characterie component M_i_ (e.g., reliability); ${\varphi }_{{B}_{i}}$ represents the attributes of other connecting components B_i_ for i = 1,2,…,B; ${RM}_{i}^{[T]}$ is the i^th^ resilience metrics over a period of time T; N is the number of total number of disruptions that may occurs during time T; ${\varphi }_{{d}_{l}}$ is the set of parameters for the lth disruption; represents the additivity of multiple disruptions	Resilience is the ability of a system to withstand potentially high-impact disruptions, and it is characterized by the capability of the system to mitigate or absorb the impact of disruption, quick recover to normal conditions	A case study was conducted using a six -machine system with two variations in configuration
Gu et al.^9^	PL^P^—production loss; ${TST}_{\varepsilon }^{P}$—throughput settling time; ${TUT}_{\varepsilon }^{P}$—total under production time.	${PL}^{P}=\frac{{t}_{D}}{{T}_{I}^{o}(0)}{PR}^{S}-\sum_{k={t}^{P}+I}^{{t}_{D}-{t}^{P}}{PR}^{P}(k)+\sum_{k=\frac{{t}_{D}}{{T}_{l}^{o}(0)}+1}^{\infty }({PR}^{S}-{PR}^{P}(k{T}_{I}^{o}(0)))$ where ${t}^{P}={t}_{R}*I\left\{P=\left.B\right\}\right.$; ${TST}_{\varepsilon }^{P}=max\left\{k|k\ge \frac{{t}_{D}}{{T}_{I}^{o}(0)}, {PR}^{P}({kT}_{I}^{o}(0))<(1-\varepsilon ){PR}^{S}\right\}{T}_{I}^{o}(0)+{T}_{I}^{o}(0)-{t}_{D}$ ${TUT}_{\varepsilon }^{P}={t}_{D}+\sum_{k=\frac{{t}_{D}}{{T}_{I}^{o}(0)}+1}^{\infty }I\left\{{PR}^{P}(k{T}_{I}^{o}(0)) < (1-\varepsilon )\left.{PR}^{S}\right\}\right.{T}_{I}^{o}(0)-\sum_{k=1}^{ {\left\lfloor {\frac{{t_{D} - 2t^{P} }}{{T_{I}^{P} (t^{{P)}} }}} \right\rfloor } }I\left\{{PR}^{P}({t}^{P}+k{T}_{I}^{P}({t}^{P})\ge (1-\varepsilon ){PR}^{S}\times \left.\frac{{T}_{I}^{P}({t}^{P)}}{{T}_{I}^{o}(0)}\right\}\right.{T}_{I}^{P}({t}^{P)}$ where PR^P^(k) is the production rate at time k under policy P (P = A, B, or O) (hereafter superscript ‘P’ is the corresponding performance under policy P); I is number of stages of the system; T_i_(k) is cycle time for each machine in stage i at time k; t_D_ is duration of the disruption; t_R_ is the time of reconfiguration; (1 − ε) is the steady-state value of production rate of the system; I{X}} is an indicator function, representing the true(1)/false(0) value of the statement X;	Resilience is the ability of a system to withstand potentially high-impact disruptions, and it is characterized by the capability of the system to mitigate or absorb the impact of disruptions, and quickly recover to normal conditions	Numerical case studies were conducted to investigate how the system resilience is affected by different design factors, including system configuration, level of redundancy or flexibility, and buffer capacities

Reference^49^ developed digital twin platform for resilience automatic analysis for reconfigurable electronic assembly line by using a systematic method based on max-plus algebra. The solution was tested in a smartphone assembly line where 6 disruptions were used to attack the system randomly. Two indicators were used for bottleneck vulnerability estimation: Vulnerability Time Delay—the time interval between the occurrence moment of a disruptive event and the moment of production stoppage at the bottleneck station and Vulnerability Time Window—the time interval between the occurrence of a disruptive event and the time point where permanent production losses occur. The resilience metric is calculated as loss of production. The calculation considers also buffers and historical information regarding potential fault modes and their repair time.

Reference^50^ assessed resilience in manufacturing plants by calculating a generic measure of POC. Inputs for calculation are the cost of the potential change (equipment investment, labor training, reprogramming, opportunity cost, and others) and the probability of change, where a ‘change’ denotes a transition from a current ‘state’ of a manufacturing system to a new state. It is relatively easy to be applied to realistic manufacturing situations. Metric was tested by a hypothetical case study in two different production systems: a 3-D printing farm and an injection molding factory, during the Covid-19 pandemic.

Reference^19^ proposes a hybrid global optimization approach to assess a service composition and optimal selection in cloud manufacturing, where resilience is one of the attributes. The inputs for resilience calculation are different attributes of endogenous and exogenous equilibriums that are finally compared with resilience that is expected from the service demander. The solution was tested by a series of experiments.

Reference^53^ developed a quantitative method for manufacturing company resilience assessment by evaluating the initial capacity loss after a disruptive event occurrence, time-dependent capacity recovery path, economic loss due to capacity reconstruction, and business interruption. The process was divided into 7 steps as follows: process flows mapping; construction of process Capacity Block Diagram; construction of Overall Reconstruction Activities Network; damage scenario definition; computation of initial capacity loss; determination of capacity recovery function; determination of economic loss.

Reference^54^ presented a quantitative resilience assessment architecture for a material handling system, including material transportation, picking, and storage. The used methods were Disruption Mode and Effects Analysis (DMEA) and the Monte Carlo method. This assessment basis on a comparison of simulation runs with and without disruption. With the tool of the DMEA system response for each disruption is found. For material handling system evaluation and modeling concerning resilience, the following input is needed: system configuration data (equipment layout parameters, system composition, equipment functional parameters), simulation-related data (iteration number, time duration, granularity of simulation), disruption-related data (disruption probability, minimum acceptable value of resilience, the shape of performance degradation curve and recovery curve). It was practically tested in a tire tread handling system, where 86 different disruption modes and disruption causes were identified.

Reference^12^ proposes probabilistic resilience metric which considers false alarm (false fault and false health) rates and reliability. A case study demonstration was carried out in an electro-hydrostatic actuator. The metric allows for estimating a system resilience more rigorously and accurately by also considering sensor faults (false alarms) in addition to the other factors.

Reference^55^ defined 3 resilience metrics: performance loss—system performance loss during the transients of a disruptive event (it can be either loss of productivity, reliability, or available functions); performance restoration time—the time the system takes to restore its throughput to a predetermined threshold and, total underperformance time—the period during which the system capacity is lower than a predetermined threshold. It characterizes both, spatial and temporal characteristics. The metrics depend on the characteristic of a disruption, system configurations, machine reliabilities, and buffer capacities. Both, temporal and spatial aspects are considered. A case study was conducted using a system comprising six production units and two variations in configuration.

Reference^9^ described three resilience metrics (throughput settling time, production loss, and total underproduction time) and analyzed them using the Bernoulli reliability model. The proposed solution was tested by a case study. The main authors overlap with^55^, therefore these two articles can be considered extensions of each other.

### AHP analysis

**Figure 8:**
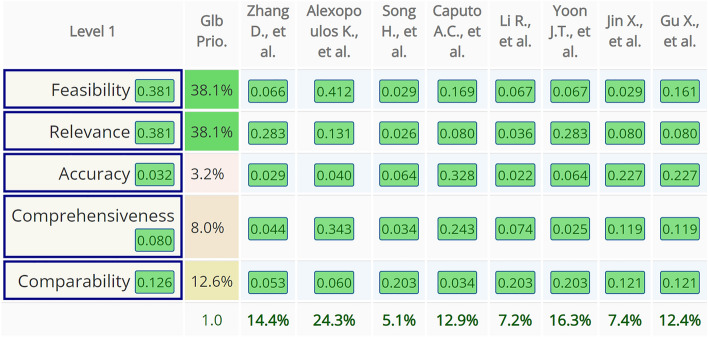
Decision matrix with comparison criteria weights, resilience metrics consolidated priority scores, and final AHP analysis results comparison (screenshot from AHP-OS software).

The AHP multi-criteria decision-making was used on a standard linear scale to compare the selected resilience metrics in five criteria: feasibility, relevance, accuracy, comprehensiveness, and comparability (Fig. 8). In pairwise comparison, the highest consistency ratio received is 4.3%, this was received from pairwise comparison under the criterion of comparability. In pairwise comparison, this level of inconsistency is allowed, and it does not influence the reliability of the results.

The article “A quantitative approach to resilience in manufacturing systems” written by Kosmas Alexopoulos, Ioannis Anagiannis, Nikolaos Nikolakis and George Chryssolouris received an AHP analysis score of 24.3% which is the highest score received. This is fresh research, published in the International Journal of Production Research in the year 2022. It proposes a methodology called POC which received the best results under the criteria of feasibility and comprehensiveness. While generic probabilistic algorithm extended with sensor faults consideration (16.3%) and max-plus algebra based systematic approach (14.4%) also received relatively higher scores compared with other metrics, whereas these articles received the highest score in the second most influencing criteria—relevance.

### POC resilience metric equations

POC is practical for manufacturing companies as it is a generic algorithm with relatively simple inputs and illustrated with a sample case study. It considers the changes related to cost which is an important factor. As manufacturing systems are considered continuous systems, there is an infinite number of potential transitions, and the changing scenario is continuous. Therefore, additionally to the main POC formula (Table 6), in a dynamic system, the POC can be calculated as follows^50^:

1 $$POC={\int }_{{X}_{1}}^{{X}_{2}}Pn\left(X\right)\,\mathrm{Pr}\left(X\right)\,dX,$$

where X_1_ is the lowest value of the potential change X, X_2_ is the highest value of the potential change X, Pn(X) is the cost distribution and Pr(X) yields the probability distribution of the potential change.

POC can be modified for different approaches. For measuring temporal penalty, the cost factor can be changed with the time factor. The main weakness of the methodology is the dependency on probabilities and related costs which decreases its accuracy.

## Discussion

The research used a literature review methodology to provide an overview of different resilience metrics and disruptions. Followed by the search string and screenings, 8 resilience metrics were identified that could be used in the assessment of resilience in manufacturing workstations. Further, the multi-criteria decision-making tool AHP pairwise comparison was applied under five weighted comparison criteria to analyze the metrics practical usage for manufacturing firms. As a result, the resilience assessment metric POC was selected as the metric with the highest value in practical usage for human-centered CPPS workstations. It received the highest score in two criteria: feasibility and comprehensiveness. A high feasibility score was the result of its clear structure and generic equation. This supports its implementation even without higher mathematics skills, which favors its wide-scale usage. High comprehensiveness score was earned by its generic structure that allows counting internal, external, human-related, and machine-related disruptions. Additionally, frequency effect sizes for extracted disruptions were found to highlight the most influencing for manufacturing companies.

According to the results, sub-RQ-s are answered as follows:

RQ_1_. Natural disasters, Covid-19, changes in the availability of materials or parts (shortage), earthquakes, terrorism, pandemics, and machine faults/breakdown are the disruptions potentially influencing manufacturing workstations the most.

RQ_2_. The existing quantitative metrics are presented in Table 5 which allow to measure the resilience of existing manufacturing workstations.

RQ_3_. POC^50^ is the most practically valuable quantitative resilience metric to assess the level of resilience in human-centered CPPS workstations in SMEs.

The research is not answering if the most mentioned disruptions have a higher rate of occurrence, more critical consequences, or the highest risk (probability multiplied by cost) for manufacturing companies. All these can be reasons for their frequent highlighting in research papers. As the collected disruptions were collected from full articles, thus many of the disruptions were collected from introductions where mostly general and topical disruptions were brought out as a list of examples. While from case studies more specific disruptions were collected. Overall, it can be still concluded that the highest frequency effect size disruptions have a relatively higher influence on manufacturing plants regardless of their core reason for mention. To generate more specific conclusions concerning mentioned reasons and dynamics, an explicitly structured data collection process should be followed.

Publishing time affects more specific disruptions, for instance newly appeared technology or diseases related disruptions. General disruptions are less influenced by timing. For instance, Covid-19 as a specific pandemic was firstly called in 2020 and received a high score, while pandemics were also mentioned in earlier years. Therefore, the list of disruptions should be considered as dynamically changing in time.

An interesting result is that Covid-19 as a specific disruption received a higher effect size than its general equivalent, while earthquake and natural disaster effect sizes are vice versa. It shows a specific pandemic higher influence on manufacturers than pandemics overall. This can be concluded by a rare rate of occurrence of pandemics as well as the high level of potential consequences if one should emerge.

Resilience can be evaluated at different levels, such as company level, manufacturing system level, and workstation level. Mostly, the compared resilience metrics were designed to use at the manufacturing system level. Many of them concentrated to assess the rearrangement possibilities of current resources using redundancy of workstations (machinery) and workers. Workstation level resilience assessment involves a more complex structure of possible solutions as it goes into more detail about specific components (sensors, actuators, etc.) while servicing subsystems (information availability, warehouse, planning) are present at both levels. Therefore, general manufacturing system-related resilience metrics can be also applied at the workstation level. In the same way, bottlenecks as critical resources can be viewed and managed at the manufacturing system level and workstation level.

POC received a high score in feasibility which reflects its practical usability. It considers the cost of changes which is an important factor as it helps to analyze and balance potential cost fluctuations. The POC is a universal metric as the cost factor can be easily changed to the time factor where needed. The weakest side of this metric is its dependency on the probability of potential changes. The metric Re^49^ received the highest score under comparison criteria relevance. It can be considered to be used in companies where digital twin is already implemented, and historical machinery-based historical datasets are collected. The other reviewed resilience metrics can be used as supportive tools for POC, for instance considering sensors false alarms or cloud manufacturing. Considering all the benefits as well as the feature that POC is a dimensionless quantity that is comparable between various workstations in a company as well as between various companies, its potential applicability in the manufacturing sector is high.

The main limitation is the accuracy of potential disruptions behavior prediction and their temporal factor. Generally, disruptions can cause unavailability of current resources (machinery faults, material shortage), instability of current resources (fluctuation of processing time, quality flaws, poor material), or potential need for a new type of resources (changing regulations, new equipment installation). Recovery of current resources covers the knowledgeable zone, which is more accurately estimable, while implementing new resources may involve an unknowledgeable zone. Therefore, flexibility is not only needed inside the manufacturing system but also in terms of openness to changes in the business environment on a broader scale. The need for new types of resources can be caused for instance by changes in legal regulations as an extra need for safety tests or by new equipment installation as a need for operators with specific knowledge and/or experience. The resilience of known resources is relatively easier to assess, while readiness for implementation of unknown resources is a more abstract feature.

Based on historical datasets, estimation of probabilities of disruptions as a function of time is well predictable at the field level mostly but leaves higher tolerance in other subcategories. An alternative approach would be to analyze the consequences of different combinations of cut-off or unstable resources (prioritizing bottlenecks), instead of focusing on certain specific probabilistic disruptions. As every disruption influences certain resources, while the number of possible disruptions is unlimited, the analysis of a limited number of disruptions may provide noncomplete or even misleading results in a sense of resilience. Therefore, research focusing on the modeling of external disruptions’ potential occurrences and their temporal behavior in manufacturing is further needed to maximize the accuracy of resilience assessment.

In our further research, POC will be used as functional input for Axiomatic Design based decomposition of resilient CPPS. This generates design guidelines for monitoring system architecture for resilient manufacturing system in digital twin perspective.

## Data Availability

The data that support the findings of this study (Supplementary Table S1 and Supplementary Fig. S1) are openly available for download in Figshare repository at https://figshare.com/articles/dataset/Analysis_of_Quantitative_Metrics_for_Assessing_Resilience_of_Human-Centered_CPPS_Workstations_Supplementary_Table_S1_and_Figure_S2/19307129 (DOI: 10.6084/m9.figshare.19307129).
